# Endotoxin clustering with allergens in house dust and asthma outcomes in a U.S. national study

**DOI:** 10.1186/s12940-020-00585-y

**Published:** 2020-03-16

**Authors:** Angelico Mendy, Jesse Wilkerson, Pӓivi M. Salo, Darryl C. Zeldin, Peter S. Thorne

**Affiliations:** 1 Department of Occupational and Environmental Health, University of Iowa, Iowa City, Iowa USA; 2 Social & Scientific Systems, Durham, North Carolina USA; 3 Division of Intramural Research, National Institute of Environmental Health Sciences, NIH Research Triangle Park, Durham, North Carolina USA; 4 University of Iowa College of Public Health, 100 CPHB, S341A, 145 N Riverside Dr, Iowa City, IA 52242-2207 USA

**Keywords:** Allergens, Asthma, Cluster analysis, Endotoxin, House dust, Wheeze

## Abstract

**Background:**

Endotoxin is ubiquitous in the environment, but its clustering with indoor allergens is not well characterized. This study examined the clustering patterns of endotoxin with allergens in house dust and their association with asthma outcomes.

**Methods:**

We analyzed data from 6963 participants of the 2005–2006 National Health and Nutrition Examination Survey. House dust sampled from bedroom floor and bedding was evaluated for endotoxin and allergens from fungi, cockroach, dog, cat, mites, and rodents. Two-step cluster analysis and logistic regressions were performed to identify the clustering patterns and their associations with current asthma and wheeze in the past 12 months, adjusting for covariates.

**Results:**

Of the homes, 17.8% had low endotoxin and allergen levels in house dust (Cluster 1). High endotoxin level clustered with *Alternaria* and pet allergens in the homes of participants with a high socioeconomic status who own pets (Cluster 2) (48.9%). High endotoxin clustered with *Aspergillus*, dust mites, cockroach, and rodent allergens in the homes of participants with low socioeconomic status (Cluster 3) (33.3%). Compared to Cluster 1, Cluster 2 was associated with higher asthma prevalence (OR 1.42, 95% CI: 1.06–1.91) and wheeze (OR 1.32, 95% CI: 1.07–1.63). Cluster 3 was positively associated with wheeze only in participants sensitized to inhalant allergens (OR 1.42, 95% CI: 1.06–1.91) or exposed to tobacco smoke (OR 1.72, 95% CI: 1.15–2.60).

**Conclusions:**

The clustering of endotoxin with allergens in dust from homes with pets or of people with low socioeconomic status is associated with asthma and wheeze.

## Introduction

Asthma is a chronic respiratory disorder characterized by bronchoconstriction, pulmonary inflammation, as well as airway remodeling with symptoms of wheezing, cough, and shortness of breath [1]. It affects over 350 million people around the world and was responsible for approximately 400,000 deaths in 2015 [2]. In the U.S., 24.6 million people currently have the condition, about 4000 people die from it every year and its annual economic cost is estimated to be over $56 billion [3, 4]. Asthma has a familial and genetic component, but home exposures are major risk factors for the development of the disease [5, 6]. We previously reported that endotoxin, a lipopolysaccharide in the cell wall of Gram-negative bacteria, was associated with a higher prevalence of asthma outcomes and chronic bronchitis or emphysema in the U.S. [7–9]. On the other hand, some reports suggest that exposure to low levels of endotoxin during early childhood could protect against allergy-mediated asthma [10]. Endotoxin is ubiquitous in our environment, but it is found at higher concentration in farms and in the homes of individuals with low socio-economic status, or in households with the presence of children, carpeting, pets, cockroaches, or with a smoker [8, 11–13].

Previous studies have indicated that most U.S. homes have simultaneous exposures to endotoxin and multiple allergens; however, the clustering of endotoxin and allergens in house dust is not well characterized [8, 12, 14, 15]. Prior reports have investigated the association of individual exposures to endotoxin or allergens with asthma and other respiratory outcomes [7–9, 16–18]. Co-exposure to multiple allergens and toxicants can also have health effects that cannot be predicted by separately evaluating the individual exposures [19]. House dust endotoxin and allergens do not exist in isolation but cluster with each other following different patterns and the association of these clusters with asthma outcomes is unknown [15]. Therefore, the aim of this study was to examine the clustering patterns of endotoxin and allergens in house dust and their association with asthma outcomes in the National Health and Nutrition Examination Survey (NHANES). The NHANES is the largest study on endotoxin and asthma to date and provides a unique opportunity to study the question [8]. It includes close to 7000 samples and measures the concentration of several allergens in homes (fungi, cockroach, dog, cat, dust mites, and rodents).

## Materials and methods

### Data source and study design

We used data from the 2005–2006 cycle of the NHANES. The NHANES is a continuous cross-sectional survey of the U.S. non-institutionalized civilian population conducted by the National Center for Health Statistics (NCHS) of the Centers for Disease Control and Prevention (CDC). It uses a complex multistage sampling design to derive a sample representative of the U.S. population. Of the 9440 total NHANES participants, 6963 dust samples were analyzed for endotoxin and allergen content. NHANES procedures and methods are further described at http://www.cdc.gov/nchs/nhanes/survey_methods.htm.

### Endotoxin and allergens analysis

Dust was collected from the bed and bedroom floor of each participant’s home with a Sanitaire™ Model 3683 vacuum cleaner and a Mitest™ Dust Collector (Indoor Biotechnologies, Inc., Charlottesville, VA). A 1-square yard (0.84 m^2^) surface on both bed and adjacent floor was independently vacuumed for 2 min. Details on the dust collection methods are available at https://www.cdc.gov/nchs/data/nhanes/nhanes_05_06/allergen_manual_06.pdf). The combined dust samples were analyzed for endotoxin content at the University of Iowa Pulmonary Toxicology Facility using a kinetic chromogenic *Limulus* amebocyte lysate assay with extensive quality assurance measures. Endotoxin concentrations were reported in Endotoxin Units (EU) per mass of sieved dust (mg) with a lower limit of detection of 0.0005 EU/mg.

The sample were also analyzed for the following allergens: fungi (*Alternaria alternata* [Alt a 1]), dog (*Canis familiaris* [Can f 1]), cat (*Feline domesticus* [Fel d 1]), dust mites (*Dermatophagoides pteronyssinus* [Der p 1] and *Dermatophagoides farinae* [Der f 1]), mouse (*Mus musculus* [Mus m 1]), and rat (*Rattus norvegicus* [Rat n 1]) using a Multiplex Array for Indoor Allergens assay (MARIA; Indoor Biotechnologies, Charlottesville, VA). Cockroach allergen (*Blatella germanica* [Bla g 1]) was assayed by enzyme-linked immunosorbent assay (ELISA) test kits from Indoor Biotechnologies, Inc. (Charlottesville, VA) and *Aspergillus fumigatus* antigen was measured by ELISA using reagents from Greer Laboratories (Lenoir, NC).

### Asthma and wheeze definitions

Current asthma was defined using the questions: “*Has a doctor or other health professional ever told you that you had asthma?*” and “*Do you still have asthma?*” Wheeze in past 12 months was defined using the question: “*In the past 12 months have you had wheezing or whistling in your chest?*”

### Covariates

Data on age, gender, race/ethnicity, and family income were collected using questionnaires. Poverty income ratio (PIR) was estimated using guidelines and adjustment for family size, year and state. Participants were also asked about housing characteristics such as the presence of a smoker in the household, mildew or musty smell, cockroaches, pets (dog and cat), carpeted floor in the home, when the house was built, and the type of home.

Serum immunoglobulin E (IgE) specific to 15 inhalant allergens was measured (*Alternaria alternata, Aspergillus fumigatus*, Bermuda grass, birch, cat dander, cockroach, dog dander, dust mites [Der p 1 and Der f 1), mouse urine proteins, oak, ragweed, rat urine proteins, Russian thistle, and rye grass. Sensitization status was defined as specific IgE against any inhalant allergen ≥0.35 kU/L.

### Statistical analysis

We first explored the correlation between house dust endotoxin and allergens by means of the Pearson method to calculate correlation coefficients (*r*). The cluster analysis was done in a two-step approach. In the first step, hierarchical cluster analysis was done using the Ward’s method to generate a dendrogram from which the number of likely clusters within our population was estimated. This estimate was then pre-specified in a k-means cluster analysis used as the principal clustering technique. The allergen concentrations in dust were log-transformed to approximate a normal distribution and standardized using z-scores. Descriptive analysis was performed and *P*-values for differences in proportions between the clusters were calculated using a chi-square test. Multivariable logistic regression was used to assess the association of each cluster with the prevalence of asthma and wheeze and odds ratios (OR) with corresponding confidence intervals (CI) were reported. The models were adjusted for age, gender, race/ethnicity, PIR, and exposure to environmental tobacco smoke (ETS). Subgroup analysis by age groups, sensitization status to inhalant allergens, and exposure to environmental tobacco smoke were performed. Effect modification was assessed by calculating the *p*-values for the interactions. All analyses were done in SAS (Version 9.4, SAS Institute, Cary, NC USA), accounting for NHANES sample weights and the complex study design in order to obtain unbiased national estimates. *P*-values < 0.05 were considered statistically significant.

## Results

**Fig. 1:**
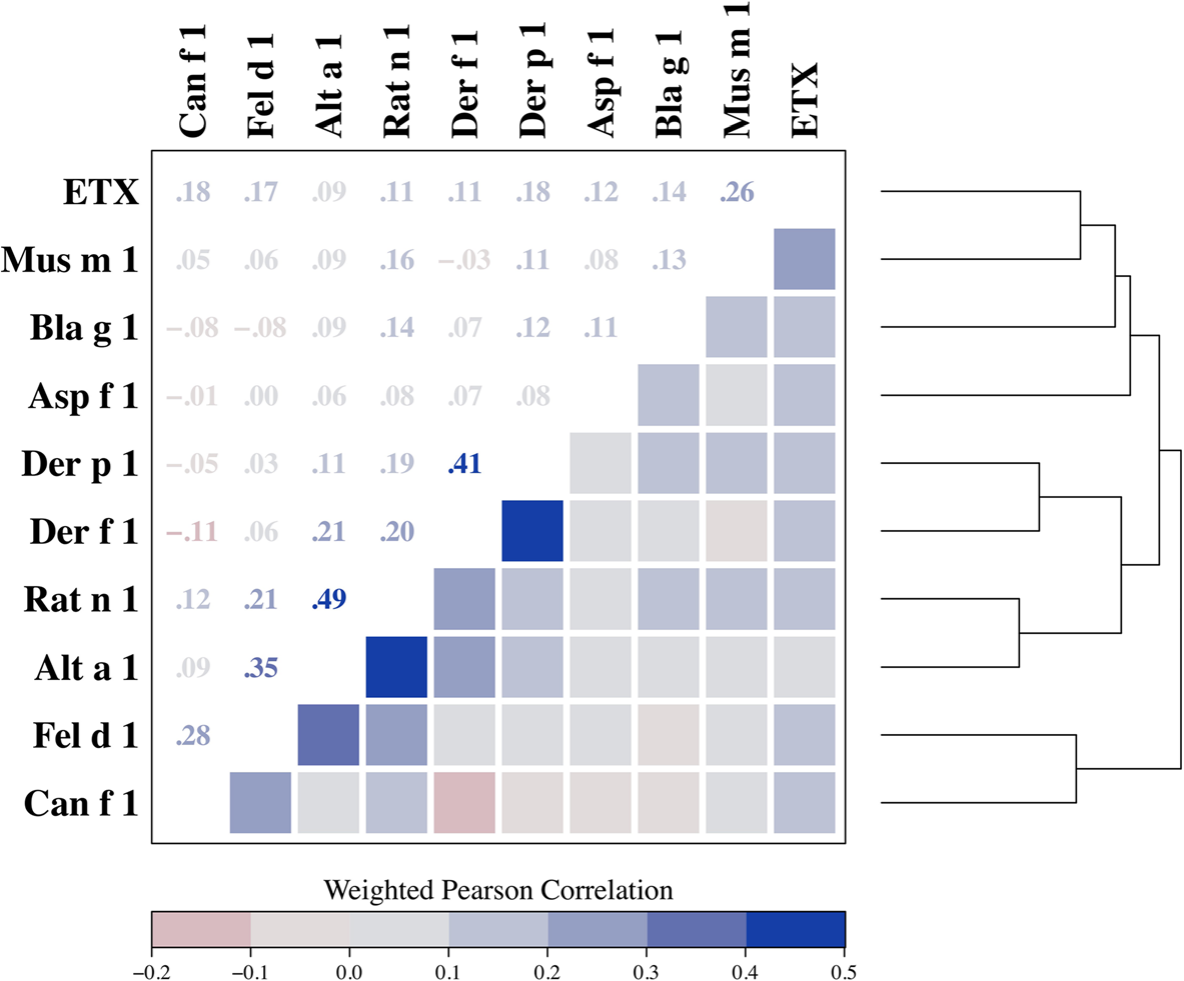
Matrix for correlation between house dust endotoxin and allergens. The dendrogram illustrates the correlation coefficient distance between endotoxin and allergens. Abbreviations: ETX, Endotoxin; Mus m 1, mouse allergen; Bla g 1, cockroach allergen; Asp f 1, *Aspergillus fumigatus*; Der p 1 and Der f 1, dust mites; Rat n 1, rat allergen; Alt 1, *Alternaria alternata*; Fel d 1, cat allergens; Can f 1, dog allergens.

Our study included 6963 dust samples collected from homes of participants who were mostly adults, non-Hispanic White, and who had a PIR ≥1.85. They also tended to live in detached family houses, in homes built before 1978, in homes without a smoker, mildew/musty smell, cockroaches, or pet (dog or cat), but with carpeted floor (Table 1). The correlation between house dust endotoxin and allergens levels as well as the dendrogram showing the correlation coefficient distance between the exposures are shown in Fig. 1. The strongest relationships were observed between *Alternaria alternata* and rat allergens (*r* = 0.49), between dust mites (Der p 1 and Der f 1) (*r* = 0.41), and between *Alternaria alternata* and cat allergens (*r* = 0.35). Endotoxin was correlated with all the studied allergens with correlation coefficients ranging between 0.09 and 0.26.

**Table 1:** Description of study participants by cluster, NHANES 2005–2006

Participants and home characteristics	Cluster 1	Cluster 2	Cluster 3	*P-value*	All participants
Prevalence, %	17.8	48.9	33.3		100
Age groups, %				**< 0.001**	
< 6 year	**5.9**	**6.1**	**8.9**		7.0
6–17 year	**9.6**	**19.9**	**17.5**		17.3
≥ 18 year	**84.6**	**73.9**	**73.5**		75.7
Gender, %				0.25	
Male	48.7	48.0	50.3		48.9
Female	51.3	52.0	49.7		51.1
Race/ethnicity, %				**< 0.001**	
Non-Hispanic Whites	**58.1**	**80.4**	**55.3**		68.1
Non-Hispanic Blacks	**23.8**	**4.1**	**18.3**		12.3
Mexican-Americans	**9.5**	**6.3**	**14.0**		9.4
Other	**8.6**	**9.2**	**12.4**		10.1
PIR, %				**< 0.001**	
< 1.85	**32.5**	**23.9**	**46.3**		32.8
≥ 1.85	**67.5**	**76.1**	**53.7**		67.2
Smoker in home, %	17.8	21.2	19.4	0.45	20.0
Mildew or musty smell, %	**14.5**	**14.3**	**21.0**	**0.006**	16.6
Cockroaches in home, %	**12.4**	**9.5**	**23.8**	**< 0.001**	14.8
Dog in home, %	**15.5**	**76.7**	**29.8**	**< 0.001**	48.0
Cat in home, %	**9.9**	**69.1**	**14.1**	**< 0.001**	36.9
Floor covering, %				0.38	
Carpeted floor	84.5	87.4	88.8		87.3
Smooth surface	11.6	9.0	7.9		9.1
Combination carpet and smooth surface	3.9	3.6	3.3		3.6
When was home built, %				**< 0.001**	
Before 1978	**41.0**	**44.2**	**57.8**		47.8
After 1978	**59.0**	**55.8**	**42.2**		52.2
Type of home, %				**< 0.001**	
Trailer	**4.8**	**6.3**	**10.4**		7.4
Detached family house	**59.8**	**73.0**	**63.0**		67.3
Attached family house	**11.0**	**8.3**	**9.0**		9.0
Apartment	**24.4**	**12.3**	**17.6**		16.2
Respiratory outcomes, %					
Current asthma	**7.2**	**10.0**	**7.8**	**0.01**	8.8
Wheezing in past 12 months	**13.6**	**18.3**	**14.3**	**< 0.001**	16.2

Abbreviations: *PIR* poverty income ratio. *P*-value for difference in prevalence between clusters calculated using chi-square test

In cluster analysis using the Ward’s method, we identified three patterns of endotoxin clustering with allergens in house dust. The levels of endotoxin and allergens in the clusters are reported in Table 2 and the characteristics of the participants by cluster and in the total study population are described in Table 1. Cluster 1 included 17.8% of the participants and defines the subgroup with low concentrations of endotoxin and allergens in house dust. This cluster included participants with a high PIR who also had the lowest prevalence of dog and cat ownership. Cluster 2 included 48.9% of the participants and labels the clustering of high endotoxin with *Alternaria*, and pet allergens. This cluster had the particularity to include participants who had a high socioeconomic status (based on PIR and living in a detached family house) and the highest prevalence of dog and cat ownership. It also had the highest prevalence of current asthma and wheeze. Cluster 3 included 33.3% of the participants and describes the clustering of high endotoxin with *Aspergillus*, dust mites, cockroach, and rodent allergens. This cluster is characterized by participants with a low socio-economic status (based on the high prevalence of PIR < 1.85 and of living in a trailer), who lived in homes built before 1978 with mildew or musty smell and cockroaches (Table 1).

**Table 2:**
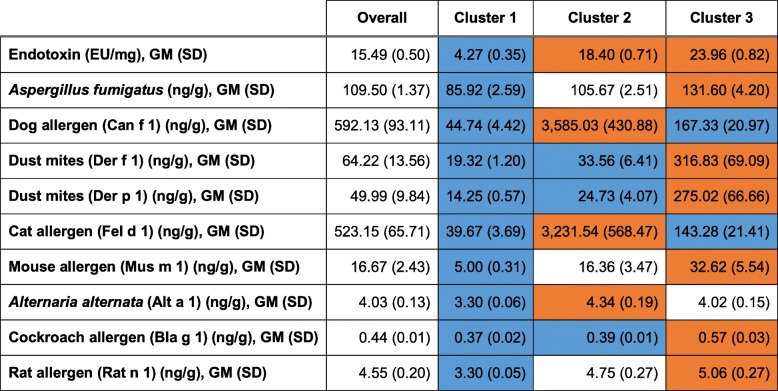
Levels of endotoxin and allergens in house dust overall and by cluster

Abbreviations: *GM* Geometric mean; *SD* Standard Deviation

**Cluster 1**: Low concentration of endotoxin and low dust allergens. **Cluster 2**: High endotoxin clustering with pet allergens (Cat and Dog) and *Alternaria*. **Cluster 3**: High endotoxin clustering with Aspergillus, Dust Mites, Mouse and Rat, and Cockroach Allergens,

Blue shading indicates endotoxin or allergen concentration significantly **lower** than the population average. Orange shading indicates endotoxin or allergen concentration significantly **higher** than the population average. *P*-value for significance < 0.05

Table 3 shows that the prevalence of sensitization to any inhalant allergen was not significantly different across clusters. However, in subgroup analysis by sensitization to specific allergens, Cluster 2 had the lowest prevalence of sensitization to dust mites and cockroach allergens among the clusters. Cluster 3 had a lower prevalence of sensitization to plant or grass allergens but had a higher prevalence of sensitization to rat allergens compared to the other clusters.

**Table 3:** Prevalence of sensitization to specific inhalant allergens by cluster, NHANES 2005–2006

Sensitization to inhalant allergens	Cluster 1	Cluster 2	Cluster 3	*P-value*	All participants
Any inhalant allergen	43.8	44.2	43.1	0.79	43.8
Any dust mite	**22.1**	**15.8**	**24.1**	**< 0.001**	19.6
Der f1	**21.2**	**13.9**	**21.5**	**< 0.001**	17.7
Der p1	**20.7**	**14.5**	**21.7**	**< 0.001**	18.0
Any pet allergen	14.9	16.6	14.9	0.50	15.7
Dog	12.0	12.0	11.2	0.84	11.7
Cat	11.3	13.1	11.0	0.42	12.1
Any plant or grass allergen	**30.7**	**29.5**	**21.7**	**< 0.001**	27.2
Ragweed	18.2	16.1	13.3	0.06	15.6
Rye grass	**22.8**	**20.4**	**16.8**	**0.0078**	19.7
Bermuda grass	**16.8**	**16.6**	**12.5**	**0.0044**	15.3
Oak	13.1	11.3	10.0	0.051	11.2
Birch	10.8	10.2	9.0	0.43	9.9
Thistle	12.8	11.8	9.3	0.053	11.2
Any mold allergen	10.3	11.0	10.9	0.85	10.9
*Alternaria alternata*	8.6	9.1	7.5	0.19	8.5
*Aspergillus fumigatus*	6.3	6.3	7.0	0.66	6.5
Any rodent allergen	1.8	1.7	2.3	0.36	2.0
Mouse	1.4	0.9	1.2	0.63	1.1
Rat	**1.0**	**1.3**	**2.1**	**0.0018**	1.5
Cockroach allergen	**11.8**	**7.7**	**12.2**	**0.0027**	9.9

Abbreviations: *Der f Dermatophagoides farinae*; *Der pDermatophagoides pteronyssinus*. P-value for difference in prevalence between clusters calculated using chi-square test

In adjusted logistic regression analysis, compared to Cluster 1 which had the lowest concentration of house dust endotoxin and allergens, Cluster 2 was associated with 42% higher prevalence of current asthma (OR 1.42, 95% CI 1.06, 1.91) and 32% higher prevalence of wheeze in the past 12 months (OR 1.32, 95% CI: 1.07, 1.63) in all participants. The association of Cluster 2 with wheeze in the past 12 months differed by age of participants (P_interaction_ = 0.002) and was only observed in adults (OR: 1.43, 95% CI: 1.15, 1.78), but not in children (OR: 0.98, 95% CI: 0.70, 1.36). The association of Cluster 3 with wheeze in the past 12 months differed by sensitization status to inhalant allergens (P_interaction_ = 0.02) and by exposure to ETS (P_interaction_ = 0.003). The relationship was seen in participants sensitized to inhalant allergens (OR: 1.42, 95% CI: 1.06, 1.91) but not in non-sensitized individuals (OR: 0.81, 95% CI: 0.61, 1.07) and in participants exposed to ETS (OR: 1.72, 95% CI: 1.14, 2.60) but not in those non-exposed (OR: 0.84, 95% CI: 0.64, 1.09) (Table 4).

**Table 4:** Association between clusters with high endotoxin and asthma, wheeze, and sensitization to inhalant allergens in all participants, NHANES 2005–2006

Clusters	Current asthma			Wheeze in past 12 months			Sensitization to inhalant allergens		
	OR (95% CI)	P	P_interaction_	OR (95% CI)	P	P_interaction_	OR (95% CI)	P	P_interaction_
***Cluster 2*versus*1***									
*In all participants*	**1.42 (1.06, 1.91)**	**0.02**		**1.32 (1.07, 1.63)**	**0.01**		1.10 (0.89, 1.34)	0.37	
*By age*									
In children	1.06 (0.62, 1.81)	0.83	0.25	0.98 (0.70, 1.36)	0.90	**0.002**	0.70 (0.42, 1.17)	0.17	**0.009**
In adults	**1.53 (1.06, 2.20)**	**0.02**		**1.43 (1.15, 1.78)**	**0.001**		1.19 (0.95, 1.50)	0.13	
*By sensitization status*									
Sensitized	1.15 (0.83, 1.57)	0.40	0.21	**1.52 (1.09, 2.13)**	**0.01**	0.49			
Non-sensitized	1.82 (0.97, 3.43)	0.06		1.24 (0.86, 1.81)	0.25				
*By exposure to ETS*									
Exposed to ETS	1.50 (0.79, 2.84)	0.21	1.00	1.54 (0.94, 2.50)	0.085	0.35	0.99 (0.54, 1.82)	0.98	0.48
Non-exposed to ETS	1.41 (0.99, 2.00)	0.06		**1.28 (1.05, 1.57)**	**0.01**		1.12 (0.95, 1.31)	0.16	
***Cluster 3*versus*1***									
*In all participants*	1.05 (0.76, 1.44)	0.78		1.01 (0.82, 1.24)	0.92		0.99 (0.86, 1.14)	0.85	
*By age*									
In children	1.10 (0.69, 1.76)	0.69	0.95	0.99 (0.69, 1.41)	0.94	0.56	0.69 (0.44, 1.06)	0.09	**0.02**
In adults	0.98 (0.66, 1.46)	0.93		1.01 (0.79, 1.28)	0.94		1.07 (0.94, 1.22)	0.32	
*By sensitization status*									
Sensitized	1.11 (0.80, 1.55)	0.52	0.83	**1.42 (1.06, 1.91)**	**0.02**	**0.02**			
Non-sensitized	1.03 (0.63, 1.68)	0.90		0.81 (0.61, 1.07)	0.14				
*By exposure to ETS*									
Exposed to ETS	1.16 (0.70, 1.93)	0.55	0.65	**1.72 (1.14, 2.60)**	**0.01**	**0.003**	0.81 (0.42, 1.59)	0.55	0.56
Non-exposed to ETS	1.04 (0.74, 1.45)	0.84		0.84 (0.64, 1.09)	0.18		1.03 (0.95, 1.12)	0.46	

Abbreviations: *OR* odds ratio; *CI* confidence interval; *ETS* environmental tobacco smoke. Odds ratios for the associations between the clusters and asthma and wheeze calculated using logistic regression. Models adjusted for age, gender, race/ethnicity, poverty income ratio, and ETS (except when the analysis was stratified by ETS)

When we examined effect modification by sensitization to specific inhalant allergens, the association of Cluster 2 with wheeze in the past 12 months was different with sensitization status to Der f 1 (P_interaction_ = 0.03) and Der p 1 (P_interaction_ = 0.02) (Supplemental Table S1). In participants sensitized to these allergens, Cluster 2 was associated with a 2-fold increase in the odds of wheeze in the 12 months (OR: 2.09, 95% CI: 1.47, 2.97 in individuals sensitized to Der f 1 and OR: 2.51, 95% CI: 1.51, 3.33 in individuals sensitized to Der p 1) (Supplemental Table S2). The association of Cluster 3 with wheeze in the past 12 months was also different with sensitization status to Der f 1 (P_interaction_ = 0.02) or Der p 1 (P_interaction_ < 0.001) (Supplemental Table S1). Cluster 3 was associated with higher prevalence of wheeze in the past 12 months among participants sensitized to Der f 1 (OR: 1.82, 95% CI: 1.07, 3.12) or Der p 1 (OR: 2.21, 95% CI: 1.30, 3.76). An inverse relationship between Cluster 3 and wheeze in the past 12 months was noted in participants who were not sensitized to Der p 1 (OR: 0.80, 95% CI: 0.65, 0.98) (Supplemental Table S2).

We tested sex of participants for effect modification to determine whether the association of the clusters with asthma outcomes differed by sex. In all participants, sex was not a significant effect modifier of the association of Cluster 2 with current asthma (P_interaction_ = 0.44), wheeze (P_interaction_ = 0.72), and sensitization to inhalant allergens (P_interaction_ = 0.60). The same was observed for the association of Cluster 3 with current asthma (P_interaction_ = 0.46), wheeze (P_interaction_ = 0.70), and sensitization to inhalant allergens (P_interaction_ = 0.63). In addition, sex was not a significant effect modifier when we stratified our analysis by the age of participants.

## Discussion

In a sample representative of the U.S. population, we performed a cluster analysis to examine the patterns of endotoxin grouping with allergens in house dust. Our results show that homes with low endotoxin often had low levels of allergens in house dust (Cluster 1). High endotoxin tended to be associated with other allergens, following different patterns. In participants with high socio-economic status who own pets, endotoxin clustered with *Alternaria* and pet allergens in house dust and this cluster (Cluster 2) was positively associated with current asthma and wheeze in the past 12 months. In participants with low socio-economic status and living in poor housing conditions, endotoxin clustered with Aspergillus, dust mites, cockroach, and rodent allergens and this cluster (Cluster 3) was only associated with wheeze in the past 12 months in those sensitized to dust mites or exposed to ETS.

Our study is the first to examine the clustering patterns of endotoxin with allergens. Previous reports explored the inter-relationship between endotoxin levels and the concentration of allergens [14]. A national U.S. study found that endotoxin levels increased with allergen burden and that endotoxin was specifically correlated with *Alternaria*, as well as with cockroach and mouse allergens [15]. These results are comparable to our findings in Cluster 3 where we reported a clustering of endotoxin with *Aspergillus*, cockroach and mouse allergens [15]. In Europe, a study found endotoxin to be positively correlated with (1 → 3)-β-D-glucans, but not with dust mites, dog, cat, and cockroach allergens [20]. In Germany, Gehring et al. reported weak but significant correlations of endotoxin with allergens from mites and cats [21]. In our descriptive analysis, the prevalence of child participants aged 6 years or younger seemed to be higher in the cluster where endotoxin levels were greater. This is consistent with previous reports that the presence of young children is a predictor of endotoxin in homes [8, 12]. Cluster 3 had the highest proportion people with a low socio-economic status which may be associated with increased levels of bio-contaminants in homes [8, 12, 22, 23]. There is also ample evidence linking low socio-economic status with the high levels of cockroach, mouse, and rat allergens in homes [24, 25]. Cluster 2 had the highest prevalence of pet ownership and had endotoxin concentrations above the population average. Pets are known predictors of endotoxin in homes, as they tend to have Gram-negative bacteria in the gut and on the skin, from which they are shed [8, 22, 26]. Cluster 2 also had high levels of *Alternaria alternata*, which is consistent with a previous analysis of the National Survey of Lead and Allergens in Housing (NSLAH) that reported that cats and dogs are predictors of this fungus in U.S. homes [27]. Participants in Cluster 3 were more likely to live in homes with mildew or musty smell or in a trailer. These participants had concentrations of endotoxin and allergens from fungus, cockroach, mite, and rodents higher than the study population average as well. In addition to being a predictor for endotoxin, indoor dampness is known to increase the risk of indoor fungal growth and exposure to mites and cockroach allergens [28, 29]. High exposure to endotoxin and mouse, cockroach allergens in trailer and mobile homes has also been reported in previous studies [30, 31].

We found the clustering of endotoxin with *Alternaria* and pet allergens (Cluster 2) was positively associated with current asthma and wheeze. In line with these results, we recently reported that exposure to cat and dog allergens enhances the relationship of endotoxin with asthma and wheeze [32]. This is consistent with previous reports that dog ownership might increase the association of pollutants with asthma [33]. However, there have been studies that found that both endotoxin as well as dog ownership might decrease asthma and allergy risk [16, 34]. It has been reported that these inverse associations were age-dependent and seem to occur in childhood during a narrow window of development [16]. This is consistent with our finding of the association of Cluster 2 with asthma outcomes only in adults [35, 36]. Regarding the exposure and sensitization to pets in relation to asthma outcomes, Gergen et al. reported that exposure to dog and cat allergens was associated with excess asthma attacks in NHANES participants sensitized to pet allergens who had asthma [37]. *Alternaria*, the other component of Cluster 2 is known to be associated with asthma outcomes [27, 38]. The clustering of endotoxin with allergens from *Aspergillus*, cockroach, dust mites, and rodent allergens (Cluster 3) was associated with wheeze in the past 12 months in participants sensitized to inhalant allergens. In line with this finding, an analysis of the National Survey of Lead and Allergens in Housing (NSLAH) data also showed that the high allergen burden was associated with asthma only in atopic participants [15]. Endotoxin, as well as every allergen of the cluster was previously reported to be independently associated with both asthma and wheeze regardless of sensitization status [27, 39–41]. However, we do not know how the exposure to all these allergens interact to affect asthma. Clusters 2 and 3 were associated in participants who were sensitized mainly to house dust mites. We previously reported that individuals sensitized to inhalant allergens are particularly susceptible to respiratory outcomes related to endotoxin alone or with other co-exposures [9, 19, 32]. Endotoxin seems to have worse respiratory effects in sensitized individuals by contributing to goblet cell hyperplasia and subsequent mucus hypersecretion that can aggravate inflammation [8, 9]. The reason specific sensitization to dust mites was a modifier of the association of clusters with asthma outcomes is unclear. However, house dust mites have been shown to increase the allergenicity of endotoxin by facilitating its interactions with TLR4 [42]. Cluster 3 was also positively associated with wheeze in the past 12 months in participants exposed to ETS. Animal models have shown that exposure to cigarette smoke increases the expression and activity of Toll-like receptor (TLR)-4, and therefore increases its sensitivity to endotoxin [43]. Matsui et al. found similar results in humans. They reported a significantly higher risk of endotoxin-related asthma outcomes in children who had high exposure to cigarette smoke [44].

Our study has some limitations. First, it was cross-sectional and the temporality between the exposures and the outcomes was unknown. Current asthma and wheeze in the past 12 months were defined by self-report and could not be confirmed. Endotoxin was sampled and measured once, but it has been suggested that endotoxin in bed and or bedroom floor stays stable for approximately a year [45]. Endotoxin was sampled in dust and it has been shown that dust endotoxin has only a modest correlation with airborne endotoxin [46]. Nevertheless, our study had major strengths. It is the first to examine the clustering patterns of endotoxin with other allergens in house dust. It includes a large sample representative of the U.S. population, which provided sufficient power to facilitate subgroup analysis on the significant effect modifiers. The endotoxin exposure assessment employed state-of-the-art techniques with extreme quality control measures. Our analysis also included a wide panel of allergens.

In conclusion, high endotoxin tends to cluster with other allergens in house dust. The clusters with high endotoxin levels are all associated with asthma or wheeze outcomes in specific subgroups of the population. We previously showed that household endotoxin decrease is effective in reducing the frequency of asthma symptoms [13]. Other studies have also examined the effectiveness of multiple trigger intervention strategies in improving asthma control [47, 48]. Future studies should evaluate whether reducing the levels of endotoxin and allergens, specifically in the homes of people who own pets and of those with low socioeconomic status, might have a greater impact in preventing asthma symptoms and perhaps reduce future incidence of other respiratory diseases such as chronic obstructive pulmonary diseases.

## Supplementary information

**Additional file 1 **: **Table S1.** Effect modification *P*-values by sensitization to specific inhalant allergens on association between clusters and asthma outcomes, NHANES 2005–2006. **Table S2.** Association between clusters with high endotoxin and asthma outcomes by sensitization to dust mites, NHANES 2005–2006.

## Data Availability

NHANES data are publicly available for data collection and analysis at https://wwwn.cdc.gov/nchs/nhanes/Default.aspx. The analytic methods and SAS Codes will be made available to other researchers for purposes of reproducing the results or replicating the procedure on request.
