# Supplementary material for: Endotoxin clustering with allergens in house dust and asthma outcomes in a U.S. national study
Source: Environ Health. 2020 Mar 16;19:35. doi: 10.1186/s12940-020-00585-y (PMC7077112; doi:10.1186/s12940-020-00585-y)
Supplement: Supplementary file 1 — Additional file 1 : Table S1. Effect modification P-values by sensitization to specific inhalant allergens on association between clusters and asthma outcomes, NHANES 2005–2006. Table S2. Association between clusters with high endotoxin and asthma outcomes by sensitization to dust mites, NHANES 2005–2006. [file 12940_2020_585_MOESM1_ESM.docx]

Supplemental Materials

**Endotoxin Clustering with Allergens in House Dust and Asthma Outcomes in a U.S. National Study**

Angelico Mendy,^a^ Jesse Wilkerson,^b^ Pӓivi M. Salo,^c^ Darryl C. Zeldin,^c^ Peter S. Thorne^a^

^a^ Department of Occupational and Environmental Health, University of Iowa, Iowa City, Iowa

^b^ Social & Scientific Systems, Inc., Durham, North Carolina

^c^ Division of Intramural Research, National Institute of Environmental Health Sciences, NIH Research Triangle Park, North Carolina

**Table S1**: Effect modification P-values by sensitization to specific inhalant allergens on association between clusters and asthma outcomes, NHANES 2005-2006

| **Sensitization** | **Current asthma** | |  | **Wheeze in past 12 months** | |
| --- | --- | --- | --- | --- | --- |
|  | Cluster 2 | Cluster 3 |  | Cluster 2 | Cluster 3 |
| Der f 1 | 0.52 | 0.10 |  | **0.03** | **0.02** |
| Der p 1 | 0.35 | **0.04** |  | **0.02** | **<0.001** |
| Cat | 0.20 | 0.08 |  | 0.83 | 0.57 |
| Dog | 0.50 | 0.34 |  | 0.86 | 0.78 |
| Cockroach | 0.39 | 0.68 |  | 0.80 | 0.37 |
| Alternaria | 0.86 | 0.61 |  | 0.98 | 0.85 |
| Aspergillus | 0.94 | 0.84 |  | 0.74 | 0.15 |
| Ragweed | 0.59 | 0.85 |  | 0.24 | 0.55 |
| Rye grass | 0.39 | 0.31 |  | 0.41 | 0.53 |
| Bermuda | 0.89 | 0.39 |  | 0.29 | 0.67 |
| Oak | 0.90 | 0.25 |  | 0.37 | 0.61 |
| Birch | 0.58 | 0.66 |  | 0.47 | 0.88 |
| Thistle | 0.95 | 0.45 |  | 0.37 | 0.65 |
| Rodent (mouse or rat) | 0.20 | 0.41 |  | 0.20 | 0.97 |

Models adjusted for age, gender, race/ethnicity, poverty income ratio, and ETS.

**Table S2**: Association between clusters with high endotoxin and asthma outcomes by sensitization to dust mites, NHANES 2005-2006

| **Clusters** | **Current asthma** | | |  | **Wheeze in past 12 months** | | |
| --- | --- | --- | --- | --- | --- | --- | --- |
|  | OR (95% CI) | P | P_interaction_ |  | OR (95% CI) | P | P_interaction_ |
| ***Cluster 2 versus 1*** |  |  |  |  |  |  |  |
| *By Der f 1 sensitization* |  |  |  |  |  |  |  |
| Sensitized | **1.78 (1.12, 2.82)** | **0.014** | 0.52 |  | **2.09 (1.47, 2.97)** | **< 0.001** | **0.03** |
| *Non-sensitized* | 1.42 (0.91, 2.19) | 0.12 |  |  | 1.21 (0.93, 1.57) | 0.16 |  |
| *By Der p 1 sensitization* |  |  |  |  |  |  |  |
| Sensitized | **1.91 (1.20, 3.04)** | **0.007** | 0.35 |  | **2.51 (1.51, 3.33)** | **< 0.001** | **0.02** |
| *Non-sensitized* | 1.36 (0.88, 2.12) | 0.17 |  |  | 1.16 (0.89, 1.50) | 0.26 |  |
| ***Cluster 3 versus 1*** |  |  |  |  |  |  |  |
| *By Der f 1 sensitization* |  |  |  |  |  |  |  |
| Sensitized | 1.60 (0.98, 2.62) | 0.06 | 0.10 |  | **1.82 (1.07, 3.12)** | **0.03** | **0.02** |
| *Non-sensitized* | 0.88 (0.54, 1.44) | 0.62 |  |  | 0.86 (0.69, 1.07) | 0.17 |  |
| *By Der p 1 sensitization* |  |  |  |  |  |  |  |
| Sensitized | 1.77 (0.98, 3.16) | 0.06 | **0.04** |  | **2.21 (1.30, 3.76)** | **0.003** | **<0.001** |
| *Non-sensitized* | 0.82 (0.53, 1.25) | 0.36 |  |  | **0.80 (0.65, 0.98)** | **0.035** |  |

Abbreviations: OR, odds ratio; CI, confidence interval. Odds ratios for the associations between the clusters and asthma and wheeze calculated using logistic regression. Models adjusted for age, gender, race/ethnicity, poverty income ratio, and ETS.
